# Recombinant Alpha, Beta, and Epsilon Toxins of *Clostridium perfringens*: Production Strategies and Applications as Veterinary Vaccines

**DOI:** 10.3390/toxins8110340

**Published:** 2016-11-21

**Authors:** Marcos Roberto A. Ferreira, Gustavo Marçal S. G. Moreira, Carlos Eduardo P. da Cunha, Marcelo Mendonça, Felipe M. Salvarani, Ângela N. Moreira, Fabricio R. Conceição

**Affiliations:** 1 Centro de Desenvolvimento Tecnológico, Biotecnologia, Universidade Federal de Pelotas, Pelotas CEP 96160-000, Rio Grande do Sul, Brazil; marcosferreiravet@gmail.com (M.R.A.F.); moreira.gmsg@gmail.com (G.M.S.G.M.); cpouey@gmail.com (C.E.P.d.C.); angelanmoreira@yahoo.com.br (Â.N.M.); 2 Curso de Medicina Veterinária, Unidade Acadêmica de Garanhuns, Universidade Federal Rural de Pernambuco, Garanhuns CEP 55292-270, Pernambuco, Brazil; marcelomendoncavet@gmail.com; 3 Instituto de Medicina Veterinária, Universidade Federal do Pará, Castanhal CEP 68740-970, Pará, Brazil; felipems@ufpa.br; 4 Faculdade de Nutrição, Universidade Federal de Pelotas, Pelotas CEP 96010-610, Rio Grande do Sul, Brazil

**Keywords:** clostridiosis, enterotoxaemia, gas gangrene, necrotic enteritis, veterinary vaccines, recombinant toxins

## Abstract

*Clostridium perfringens* is a spore-forming, commensal, ubiquitous bacterium that is present in the gastrointestinal tract of healthy humans and animals. This bacterium produces up to 18 toxins. The species is classified into five toxinotypes (A–E) according to the toxins that the bacterium produces: alpha, beta, epsilon, or iota. Each of these toxinotypes is associated with myriad different, frequently fatal, illnesses that affect a range of farm animals and humans. Alpha, beta, and epsilon toxins are the main causes of disease. Vaccinations that generate neutralizing antibodies are the most common prophylactic measures that are currently in use. These vaccines consist of toxoids that are obtained from *C. perfringens* cultures. Recombinant vaccines offer several advantages over conventional toxoids, especially in terms of the production process. As such, they are steadily gaining ground as a promising vaccination solution. This review discusses the main strategies that are currently used to produce recombinant vaccines containing alpha, beta, and epsilon toxins of *C. perfringens*, as well as the potential application of these molecules as vaccines for mammalian livestock animals.

## 1. Introduction

*Clostridium perfringens* is Gram-positive, rod-shaped, spore-forming, and anaerobic (but not strictly anaerobic) [1]. This commensal bacterium is ubiquitous in the gastrointestinal tract of healthy humans and animals [1,2,3]. To date, it is known that *C. perfringens* produces up to 18 toxins: alpha (CPA), beta (CPB), epsilon (ETX), iota (CPI), enterotoxin (CPE), theta/perfringolysin O (PFO), beta-2 (CPB2), TpeL, NetB, NetF, BecA/B, NanI, NanJ, kappa, mu, lambda, α-clostripain, and delta toxin [4]. CPA, CPB, ETX, and CPI are used to group *C. perfringens* into five toxinotypes (A–E) according to the toxins that the bacterium produces [5]. Each toxinotype causes different diseases in a range of farm animals and humans [6].

There are two main routes by which the *C. perfringens* can infect animals and humans. The iatrogenic or traumatic route is exclusive to *C. perfringens* toxinotype A, and causes gas gangrene or malignant edema. The second route is by ingestion of spores or through changes in the gut flora followed by excessive growth of *C. perfringens* and toxin production, which causes enterotoxemia, necrotic hemorrhagic enteritis, and enterocolitis [1,6,7,8]. This second route of infection is common to all toxinotypes. Diseases caused by CPA, CPB, and ETX are responsible for significant economic losses throughout the world due to their high lethality rates and because they affect farm animals that have a high zootechnical performance (Table 1) [6,9]. In Brazil only, clostridiosis are the cause of death of approximately 500,000 bovines per year, resulting in a loss of about US$350 million [10].

*C. perfringens* infections can be treated through the administration of penicillin G, hyperbaric oxygen, and monoclonal antibodies, or through the surgical removal of affected tissues [12,13,14,15]. Although not completely effective in humans, these treatments are an alternative. However, for farm animals, these options are usually not viable, since death occurs very quickly [12]. Therefore, prophylactic measures need to be employed to fight *C. perfringens* infections. Vaccination against CPA, CPB, and ETX currently represents the best prophylactic measure. Toxoids are obtained from growing *C. perfringens* and inactivating the toxins using formaldehyde [16,17]. Even though toxoids induce the production of neutralizing antibodies, they present some drawbacks. For example, they are associated with a risk of residual toxicity due to incomplete formaldehyde inactivation and a risk of residual formaldehyde being present [18,19,20]. Furthermore, the potency of the vaccine varies from batch to batch [17,21,22]. In addition, the toxoids that are produced from the supernatant of *C. perfringens* cultures are not pure (i.e., other toxins and proteins are present), and this accounts for a higher degree of antigen diversity in the vaccine [23]. Finally, growing *C. perfringens* poses a significant biosafety risk and requires the application of strict biosafety measures.

More than 150 million clostridial toxoid doses are produced annually in Brazil [22]. The rate of production of the clostridial vaccine is increasing on an annual basis as the number of farm animals continually rises. At present, it is impossible to eradicate *C. perfringens*. Recombinant versions of CPA, CPB, and ETX, which are produced mainly in *Escherichia coli*, have gained ground as promising alternatives to the clostridial vaccine because they present solutions for the aforementioned issues. The use of non-pathogenic *E. coli* strains and the fact that recombinant toxins may present reduced or no toxicity can minimize, or even obliterate, some of the problems associated with native toxin production. Furthermore, heterologous protein expression in *E. coli* can be tightly controlled, allowing the uniform production of recombinant toxins once the culture conditions have been optimized. Because some recombinant toxins have barely any toxicity, there is no requirement for the use of formaldehyde, which makes the production process simpler and safer. Finally, yet importantly, growing *E. coli* is a less laborious process than cultivating *C. perfringens*.

Recombinant proteins in *E. coli* are expressed in different amounts either as: (1) a soluble protein; (2) an insoluble protein; or (3) a mix of both forms depending on the protein itself. *E. coli* expression strain, expression vector, coding DNA and protein sequences, growth conditions (e.g., temperature, medium, etc.), and inductor concentration can influence the amount and solubility of the recombinant protein [24,25]. Both soluble and insoluble forms of recombinant proteins present pros and cons from the perspective of vaccine production. It is highly likely that the soluble recombinant proteins present the same 3D conformation as the native protein, maintaining the conformational epitopes that might be crucial to confer immunity. A further advantage of soluble recombinant proteins obtained in the cell lysate supernatant (CLS) is that they do not require denaturing for purification, requiring only the removal of other proteins and LPS, which can be performed by a combination of different chromatography techniques (e.g., affinity, ion exchange, size exclusion, etc.). Insoluble proteins, on the other hand, may achieve higher levels of purity as inclusion bodies can be washed several times. Furthermore, different chromatography techniques can also be employed for insoluble proteins provided they go through denaturation and refolding steps. However, insoluble proteins may lack conformational epitopes that are crucial for immunity, and refolding steps do not guarantee that these epitopes recovered on the protein structure.

In recent years, researchers have invested significant energy in developing more industry-friendly methods of producing recombinant proteins. Non-purified inclusion bodies and CLS have been tested as immunogens against *C. perfringens* toxins in both experimental and farm animals [20,22,26]. Moreira Jr et al. [27] immunized guinea pigs with inclusion bodies and CLS, and they also validated this strategy against botulinum toxins serotypes C and D. Their results enhanced the appeal of non-purified recombinant proteins for use in veterinary vaccine industries. The aim of the current review was to examine the most frequently used strategies to produce recombinant CPA, CPB, and ETX toxins. Additionally, this review accesses the protective potential of the recombinant toxins produced under different conditions for mammalian livestock animals.

## 2. Alpha Toxin (CPA)

CPA is coded by the chromosomic gene *plc*. This gene is present in every toxinotype and is expressed at the highest levels in toxinotype A [28]. The active toxin CPA is a 370-residue long, zinc-dependent, phospholipase C (PLC) with sphingomyelinase and lectinase activity and approximately 42.528 kDa [29,30]. The LD_50_ for CPA has been calculated as 3 µg/kg in mice [11]. This toxin is divided into the *N*-terminal (1–246) and the *C*-terminal (247–370) domains. The *N*-terminal domain comprises the catalytic core of the toxin, while the *C*-terminal domain and the central loop (55–93) are responsible for binding host phospholipids and GM1a ganglioside respectively (for review, see Oda et al. [31]). Two zinc ions (Zn^2+^) are strongly bound to CPA. One ion is bound to His148 and Glu152 and is crucial for enzymatic activity. The remaining ion is bound to His11 and Asp130 and has structural function [32,33]. Histidine residues 68, 126, and 136 also bind calcium and help the toxin bind phospholipids in the host cell membrane.

CPA is responsible for intravascular hemolysis, platelet aggregation, and capillary damage. These factors stop leukocytes and oxygen from getting to the site of infection and create an environment that is favorable for the proliferation of *C. perfringens*. In gas gangrene cases, CPA helps immune evasion by interfering in neutrophil migration to the infected tissue, minimizing the number of mature cells in the bone marrow, and causing the accumulation of neutrophils in adjacent vessels [34].

### Recombinant CPA Production Strategies and Animal Model Immunizations

The first studies involving the cloning and expression of the gene encoding CPA determined the nucleotide sequence and protein molecular weight of CPA [35,36,37,38,39]. This way, a 28-amino acid *N*-terminal signal peptide was identified, as well as some biochemical properties of the toxin were described. Many strategies have been employed in an attempt to obtain a non-toxic version of CPA for vaccination, the most common of which are site-directed mutagenesis, isolation of the strains that naturally produce non-toxic CPA, expression of only the *N*- or *C*-terminal domain, expression of chimeric toxins, and expression on the surface of *Bacillus subtilis* spores (Table 2) [32,40,41,42,43,44,45].

Site-directed mutagenesis studies initially aimed to characterize essential residues for CPA toxicity and became the base for the production of genetically modified, non-toxic, immunogenic, recombinant CPA. It has been reported that the H68G, H148G/L, D56G, and E152Q mutations are capable of obliterating the toxicity of CPA [32,33]. In one study, mutation of D56N was able to reduce platelet aggregation and PLC activity, thereby increasing LD_50_ from 0.5 to 100 μg/kg in mice [49]. The researchers found that the mutation T272P reduced CPA toxicity by 35% [50]. Site-directed mutagenesis of D336N, Y275N, D269N, Y331L, Y331F, Y307F, and Y275F reduced hemolytic activity by 11%, 11%, 19%, 30%, 36%, 38%, and 73%, respectively [51]. Shoepe et al. [45] identified a naturally occurring non-toxic variant of CPA (CPA-121A/91) with M13V, A174N, T177A, H212R, P295Q, S335P, I345V, and W360G mutations. This variant presented no hemolytic, PLC, or sphingomyelinase activity. In an alternative study, vaccinations with CPA-121A/91 were able to extend the lifespan of challenged mice, but could not prevent death. Interestingly, reversion of the H212R mutation was able to protect 76% (17/21) of the vaccinated mice [52]. These results provide some insights into the possible epitopes and crucial sites for the toxin to act, and might prove useful for the development of both protective and therapeutic antibodies.

Recombinant, non-mutated CPA may present residual toxicity with dermonecrotic activity and might not be suitable for vaccination [53]. Formaldehyde is extensively used to detoxify native CPA, although it also reduces immunogenicity [19,54,55]. Alternatively, site-direct mutagenized recombinant or naturally occurring non-toxic CPA could be used for vaccination; however, in such cases, the immune response will be against the whole toxin, not solely the protective epitopes. Immunization of mice with *N*- and *C*-terminal domains of CPA (CPA-N^(1–246)^ and CPA-C^(247–370)^, respectively), or with the latter fused to GST (GST-CPA-C^(247–370)^) expressed in *E. coli* demonstrated that CPA-C^(247–370)^ (19 kDa) alone is capable of conferring immunity against challenge with 50 µg of CPA or 10^9^ *C. perfringens* cells [44,48]. Animals inoculated with CPA-N^(1−246)^ were not protected against CPA. Taken together, these results indicate that blocking CPA binding to host cell is a necessary and sufficient method of conferring immunity against this toxin. It negates the need to neutralize its enzymatic activity and renders the *C*-terminal domain as the main vaccine candidate against CPA. Furthermore, in one study, mice vaccinated with rCPA-C^(247–370)^ were protected against PLCs from *Clostridium absonum* (CAA) and *Clostridium bifermentans* (Cpb), which share 60% and 50% identity respectively with *C. perfringens* CPA [46].

**Figure 1:**
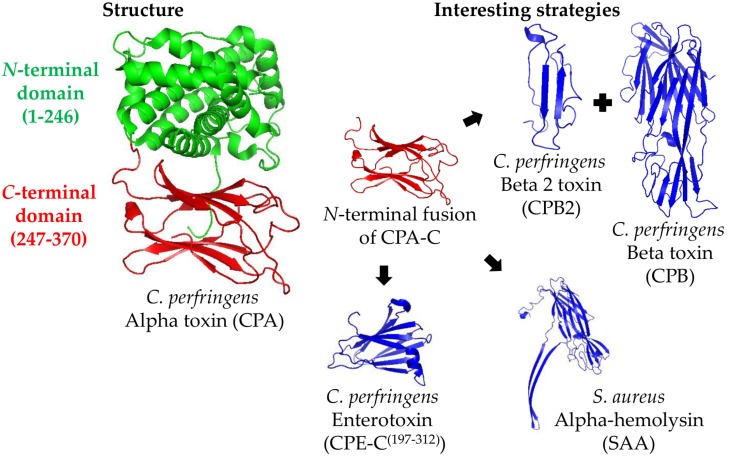
Representation of the *Clostridium perfringens* Alpha toxin (CPA) structure and the main strategies for its production as a recombinant antigen. The CPA structure (PDB ID: 1CA1, left part) is divided in the *N*-terminal (amino acids 1–246, in green) and *C*-terminal (amino acids 247–370, in red) domains. The most interesting strategies to use CPA as a vaccine antigen consist on using its *C*-terminal domain (CPA-C) fused to the *N*-terminal part of other antigens, such as *C. perfringens* Beta 2 toxin (CPB2) and Beta toxin (CPB), *Staphylococcus aureus* Alpha-hemolysin (SAA; PDB ID: 3ANZ), or *C. perfringens* enterotoxin *C*-terminal domain (CPE-C; PDB ID: 2XH6) (right part).

Structural vaccinology is a branch of structural biology that studies the epitopes responsible for conferring immunity. It is possible to design chimeras that consist only of protective epitopes of different toxins and to exclude the domains that do not confer immunity. This approach simplifies the production process because only one process is required to produce a chimera that can confer immunity to a range of toxins as opposed to many processes being executed for different toxins [56]. Considering this, modifying the whole rCPA molecule excluding unnecessary domains would be a useful approach. In fact, the rCPA-C^(247–370)^ domain is being used to replace the whole rCPA as vaccine component, allowing the construction of novel chimeras for experimental vaccines against *C. perfringens*. For example, a recombinant chimera (rCPAE) comprising CPA-C^(284–398)^, fused to the *C*-terminal portion of *C. perfringens* Enterotoxin (CPE-C^(197–312)^), was found to protect 100% (12/12) of mice challenged with CPA, and 75% (9/12) of mice challenged with CPE [43]. The protection induced rCPAE face the challenge with both CPA and CPE toxins has not been evaluated. CPA-C^(284–398)^ fused to the *N*-terminal domain of *Staphylococcus aureus* Alpha-hemolysin (SAA^(36–221)^) protected 100% (6/6) of mice challenged with either CPA or SAA, and 81.3% (5/6) of mice challenged with both toxins (Figure 1) [41].

Zeng et al. [26] evaluated four vaccine formulations against *C. perfringens* toxins: (1) rCPA; (2) bivalent recombinant chimera comprised of CPB and CPB2—rCPB2B1; (3) co-administration of rCPB2B1 and rCPA; and (4) trivalent recombinant chimera comprised CPA, CPB, and CPB2—rCPAB2B1. The recombinant antigens were expressed and used as inclusion bodies in immunizations. Mice vaccinated with rCPA presented 80% protection (24/30) when challenged with 1 × LD_100_ of *C. perfringens* toxinotype C culture supernatant. Group 3 was 100% (30/30) protected against twice the challenge dose of Group 1. Group 4 was 93% (28/30) protected against the same challenge. The authors argued that the lower protection observed in Group 4 in comparison to the groups that received co-administered antigens was due to an alteration in conformational epitopes that resulted from many antigens joining together in only one polypeptide chain. Goossens et al. [53] demonstrated that animals inoculated with GST-CPA-C^(247–370)^ were less protected than animals inoculated with CPA-C^(247–370)^ alone, suggesting that the presence of GST disrupts the protective potential of the *C*-terminal domain of CPA. Williamson and Titball [44] previously obtained similar results when mice vaccinated with CPA-C^(247–370)^ produced two times as many neutralizing antibodies as mice vaccinated with GST-CPA-C^(247–370)^. Surprisingly, the GST-CPA-C^(247–370)^ chimera expressed on the surface of *B. subtilis* spores elicited the production of both systemic IgG and sIgA in the saliva, feces, and lung samples of the vaccinated animals. Mice immunized with 2 × 10^9^ or 5 × 10^10^ orally or intranasally respectively, were 100% (6/6) protected against 12 × LD_50_ [40]. These results suggest that it is not just vaccine composition and antigen design that are essential to the generation of immunity, but also the fashion in which antigens are presented to the immune system is crucial to achieving immunity against high doses of challenge. We strongly suggest all these aspects are taken into account when designing and testing novel vaccines, not only for clostridial toxins but also for all pathogens.

*E. coli* is by far the most used expression system for the expression of rCPA. Two kinds of plasmid vectors are frequently employed for this purpose: pT7, and pET. Both vectors contain the T7 promoter, an antibiotic resistance gene, and a copy of the *lacI* gene for the regulation of the expression. The *E. coli* strain BL21 (DE3), which contains the coding gene for the T7 DNA polymerase in its genome under the control of lac operon, is the most commonly used strain. Lactose or similar molecules, such as alollactose or the synthetic derivate of galactose isopropyl-β-1-d-galactopiranoside (IPTG), which cannot be metabolically degraded by *E. coli*, can induce the lac operon. For the expression of rCPA, concentrations of 0.3–1 mM of IPTG are described in the literature as successful, and, most interestingly, only one work has described the attainment of an insoluble protein, although the culture conditions (i.e., medium, temperature, and induction time) were very similar to other works that described soluble rCPA. The expression of insoluble antigens is always perceived to be a problem for recombinant vaccine development since many protective epitopes can be lost due to erroneous protein folding. Thus, the optimization of expression conditions (medium, inductor concentration, pre-induction, and induction time, etc.) is often indicated. However, Zeng et al. [26] described how the inclusion bodies of rCPA can be successfully used for animal vaccination without the need for denaturation, refolding, or even purification.

## 3. Beta Toxin (CPB)

CPB is coded by the plasmid-encoded gene *cpb* and is a member of the heptameric proteins family that is known as beta-pore-forming toxins (BPFT) [57]. Expression of this gene results in a protoxin with 336 amino acids. When it is secreted, a 27-amino acid signal peptide is removed, resulting in the active toxin with 34.861 kDa. This toxin has the ability to form oligomers in vitro, an activity that allows it to develop cation-selective channels of approximately 228 kDa and 12 Å diameter within the lipid microdomains composed of phosphatidylcholine and cholesterol in the plasma membrane [58]. The LD_50_ for CPB in mice is 0.4 µg/kg, and its toxicity is characterized by a fatal necrosis without hemolysis [11]. This toxin is also sensitive to trypsin, which can completely inhibit its activity. As such, newborn animals are at risk of being infected by CPB-mediated disease as they produce low amounts of gastric proteases and colostrum also contains trypsin inhibitors [6,59].

### Recombinant CPB Production Strategies and Animal Model Immunizations

Early studies of the *cpb* gene from *C. perfringens* type B strain NCTC8533 described its identification, sequencing, and cloning into the pBET7 vector for expression on *E. coli* JM109. The protein exhibited approximately 34 kDa and reacted against antibodies raised for the native CPB [60]. Further studies have focused on determining the toxicity mechanism of CPB and have generated important recommendations for vaccinology specialists that have facilitated the development of recombinant vaccines [61,62,63,64]. Of the various strategies that are used to develop recombinant vaccines containing CPB, four approaches, in particular, are worth highlighting: (1) the insertion of point mutations for the generation of toxoids; (2) the expression of the whole toxin sequence; (3) the expression of its *C*-terminal domain (CPB-C^(143–311)^); and (4) the expression of chimeric antigens containing other toxins (e.g., CPA, CPB2, or ETX) or the B subunit of the heat-labile enterotoxin of *E. coli* (LTB) (Table 3) [18,26,65,66,67,68,69,70,71].

In terms of the first mentioned approach to generating possible vaccine candidates, it is known that the Y203F, R212E, and R121Q mutations are able to increase the LD_50_ by CPB 2.5, 12.5, and 5.5 times respectively [65,66]. However, Nagahama et al. [72] obtained a non-toxic rCPB molecule by introducing the Y266A, L268G, and W275A mutations in combination, or by substituting C265 for Tyrosine or Histidine. However, none of these constructs were evaluated in terms of their ability to generate protective antibodies when injected into animal models.

One successful study that used the whole toxin sequence was performed by subcloning the coding sequence of CPB into the pAE vector for expression in *E. coli* BL21 (DE3) Star^TM^ [67]. The resulting protein was obtained in inclusion bodies, which had to be suspended with urea. After purification, a refolding step was conducted using PBS, and this resulted in a completely non-toxic protein. In this case, this protein was able to generate 10 IU/mL of protective antibodies when 100 µg of rCPB was injected in rabbits. Although the refolding step is usually considered a problem, it is possible that it plays an important role in reducing or eliminating the toxicity of the protein, as was the case in this study. On the other hand, other options, such as using the inclusion bodies directly (without purification and further refolding) have proven to be successful for rCPA and rETX in terms of both maintaining the non-toxicity of the proteins and generating high levels of protective antibodies [22,26]. If there is a necessity to eliminate the refolding step, the direct use of the rCPB inclusion bodies appears to represent a reasonable approach to doing so.

By analyzing the sequence of CPB, it was determined that its *C*-terminus residues (CPB^(256–276)^) are closely related to the *C*-terminal part of alpha toxin from *S. aureus* (SAA^(245–267)^) [72]. This supports the finding that CPB^(256–276)^ has a similar function and, therefore, it is responsible for binding to the cell receptor. As is the case with other toxins, such as botulinum neurotoxins, the receptor-binding domain usually contains the major part of the protective epitopes. An in silico study determined three possible B-cell epitopes in CPB (amino acids 32–45, 140–156, and 260–275) [18]. However, although the epitope CPB^(140–156)^ was fused to the LTB molecule for further characterization, none of these epitopes were tested in experimental vaccines, leaving an open space for the investigation of minor regions of the protein that can lead to protective immune responses.

**Figure 2:**
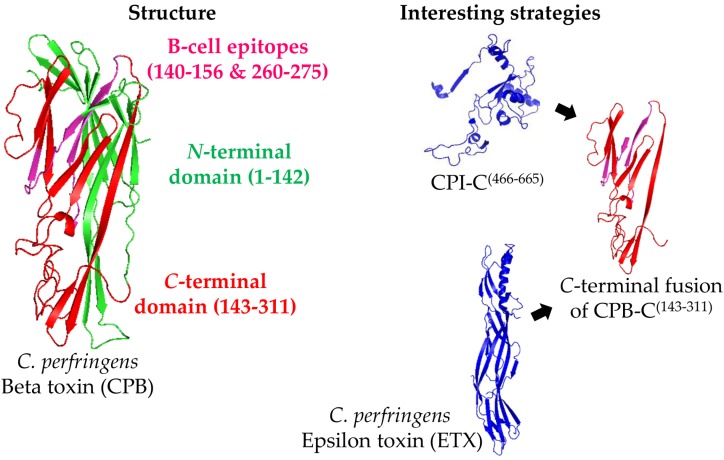
Representation of the *Clostridium perfringens* Beta toxin (CPB) structure and the main strategies for its production as a recombinant antigen. The CPB structure was predicted by SwissModel online software (Genbank ID: L13198) using standard settings (left part). Since the use of the *C*-terminal domain of CPB (CPB-C) seems to be the most promising option for vaccine development, two predicted B-cell epitopes are shown (magenta), although they have not yet been tested as vaccine. The most interesting strategies to use CPB as a vaccine antigen consist on using its *C*-terminal domain (amino acids 143–311; CPB-C, in red) fused to the *C*-terminal part of other antigens, such as the *C*-terminal domain of *C. perfringens* Iota toxin (CPI-C), or *C. perfringens* Epsilon toxin (ETX; PDB ID: 1UYJ) (right part).

Bearing in mind the fact that epitopes have yet to be tested as vaccine candidates, the use of whole proteins or smaller parts of the protein fused to other antigens is also a strategy that is in need of further exploration (Figure 2). A study with the region CPB^(143–311)^, which was fused to the *C*-terminal part of CPI (CPI-C^(466–665)^), showed that the resulting protein (called rCPIB) was able to protect 83% and 91% of mice challenged with 5 × LD_100_ of CPI and CPB, respectively [70]. Similarly, Bai et al. [73] produced a bivalent chimera that contained CPA and CPB (rCPAB), which was able to protect 100% (10/10) of mice challenged with 1 L+ of CPB. Another chimeric antigen with ETX and CPB (rETXCPB) was able to generate titers of 6 and 10 IU/mL against the respective toxins in rabbits [71]. A similar approach that employed two variants of CPB (chimera rCPB2B1) was able to protect 90% (27/30) of mice challenged with 1 × LD_100_ of culture filtrated of *C. perfringens* toxinotype C [26]. In the same study, the co-injection of rCPA plus rCPB2B1 resulted in the 100% (30/30) protection of the challenged mice. This last result indicates that these three toxins, CPA, CPB, and CPB2, have a synergistic effect on the pathogenesis of toxinotype C.

One relevant aspect to the heterologous expression is that of codon optimization. The majority of the time, the use of optimal codons for *E. coli* expression facilitates the attainment of a higher protein yield; however, there is no guarantee that a soluble protein will be obtained. Sakurai and Nagahama [59], for example, obtained an insoluble rCPB even when using *E. coli* optimal codon. The existing literature describes how it is possible to overcome this problem through the use of a chimeric antigen to improve the solubility of the antigen. As described previously, both the rETXCPB and rCPIB contain CPB, or part of it, in the *C*-terminal region, having soluble proteins at the end [70,71]. However, in the same study, both the rCPAB2B1 and the rCPA that were produced during the research were insoluble [26]. Further studies have found that the strain of the *E. coli* can influence the solubility of the protein. For example, Bakhshi et al. [74] found that the rCPB expressed in BL21 (DE3) was insoluble, while that expressed in Rosetta (DE3) was soluble. Reducing the expression temperature (to between 16 and 28 °C) is a common approach by which researchers try to increase protein solubility. However, the use of this method for the expression of rCPB has not been described in the literature.

## 4. Epsilon Toxin (ETX)

ETX is coded by the plasmid-encoded gene *etx*, and is the third most potent toxin known from *Clostridium* spp., behind botulinum toxins (BoNTs) and tetanus toxin (TeNT) (for review, see Alves et al. [75]). This toxin is part of the pore-forming aerolysin-β-toxins, and is produced by the B and D toxinotypes. ETX is translated as a 32.98 kDa protoxin that is then activated by trypsin and chymotrypsin proteases. It is also thought that λ-protease from *C. perfringens* itself may also activate ETX in some strains [76,77,78]. An ex vivo study on part of a caprine intestine found that the activation of ETX is more complex than initially believed [79]. The active toxin was known to have about 27 kDa based on SDS-PAGE. However, MS analysis showed products with 27.688, 27.801, and 27.900 kDa with divergences on the residues of the *C*-terminal region. This suggested that carboxypeptidases other than trypsin and chymotrypsin could activate ETX [79]. These findings were very important for further studies on the activity of the toxin, since the LD_50_ of the toxin in mice varies from 50 to 320 ng/kg depending on the protease used for activation. For example, the combination of trypsin and chymotrypsin can result in the maximum activation of the toxin, reaching the LD_50_ with 50 ng [76,77,78].

ETX contains three domains: domain I is responsible for the interaction with the host cell receptor, domain II stabilizes the interaction of the toxin with its receptor and triggers the heptamerization, and domain III is responsible for the interaction between the monomers for the formation of the pore on the membrane [75]. Y29, Y30, Y36, Y196, and F199 from domain I are the main amino acids involved in the interaction with the cell surface [80,81]. A further detail about the activity of the toxin that is of significance is that the mutation Y196E and the presence of the 23-amino acid *C*-terminal peptide act mutually to reduce the toxicity of ETX. This *C*-terminal peptide impairs the binding of the toxin to the cell, while the Y196E mutation eliminates the oligomerization and, thus, the pore formation. Jiang et al. [82] evaluated the role Y71 (domain III) plays in ETX activity, and found that the lack of this amino acid eliminates the toxicity when tested in vitro on MDCK (Madin-Darby canine kidney) cells.

Another study on the interaction of ETX with the plasma membrane of MDCK cells found that the hepatitis A virus cell receptor 1 (HAVCR-1), which is present in detergent-resistant microdomains, is the target of ETX [77,83]. Rumah et al. [84] showed binding of ETX to its target cells and its cytotoxic activity on mammalian cells are dependent on myelin and lymphocyte (MAL) protein. The pores formed by ETX include seven monomers of the toxin and some other proteins from the cell membrane, such as caveolin-1 and -2. The whole complex shows around 700 kDa and allows the Na^+^ and Ca^2+^ ions to go inside the cell, resulting in ATP depletion. Furthermore, ETX also increases the permeability of the mitochondrial membrane, causing a rapid transport of the caspase-independent apoptosis factor AIF to the nucleus [85,86,87]. As such, the toxin causes intestinal necrosis, through which the GAP junctions of the enterocytes are rapidly disrupted, allowing ETX to reach the bloodstream and, consequently, other organs such as kidneys, lungs, liver, and the brain [88].

### Recombinant ETX Production Strategies and Animal Model Immunizations

The first study that assessed the cloning, sequencing, and expression of ETX in *E. coli* served as a base for further studies that sought to determine its structure, toxicity, interaction with host cells, and immunogenicity [22,82,89,90]. The recombinant toxin was used to determine the toxicity mechanism, the main susceptible cell types, preferential organs, and potential hosts [78,91,92,93].

A vast number of studies have focused on ETX due to the fact it is the third most potent toxin know and, therefore, is considered a potential biowarfare threat according to the Centers for Disease Control and Prevention (CDC), USA [94]. Even though it is highly lethal to humans, ETX is more prevalent in cases of enterotoxemia in ovine, caprine, and bovine. This disease also acts rapidly and has a high fatality rate. Since treatment is not a feasible option, this increases the need for a prevention method, mainly via vaccination, that can avoid the loss of animals. The main approaches for the development of a recombinant vaccine against ETX are the use of rETX-carrying mutations to eliminate its toxicity, the use of *Lactobacillus casei to* carry these mutated antigens to its surface, the expression of the protoxin (rPETX), and the use of chimeras that contain multiple antigens (Table 4) [20,22,69,71,80,95,96,97,98].

In its structure, ETX has two 35-amino acid parallel strands of β-sheets that cross all its structure, passing through domains I, II, and III [99]. For this reason, the use of only one of the domains as a vaccine candidate, what is the case for both CPA and CPB, is still a challenge. Considering this, the current strategies by which rETX is produced for vaccine applications are focused on the development of a non-toxic molecule that is able to generate protective immunity. Thus, many mutated antigens have been developed and tested as vaccine candidates in animal models.

**Figure 3:**
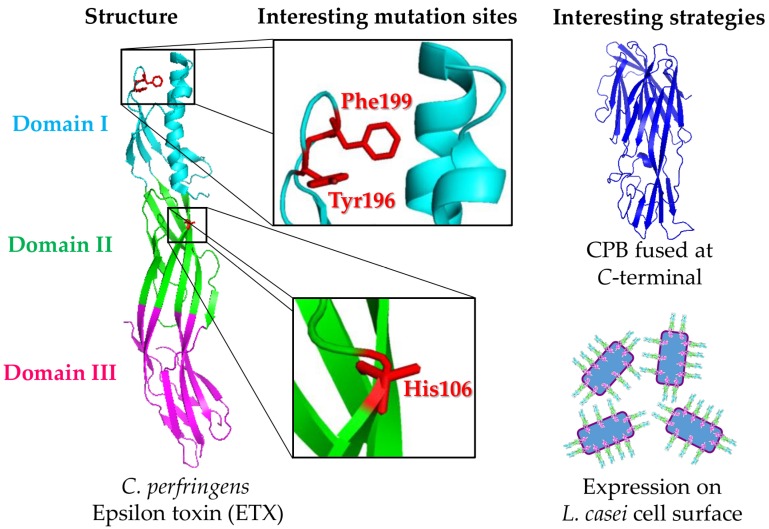
Representation of the *Clostridium perfringens* Epsilon toxin (ETX) structure and the main strategies for its production as a recombinant antigen. The ETX structure (PDB ID: 1UYJ, left part) is divided in three domains, named I, II, and III (cyan, green, and magenta, respectively). Because ETX has two 35-amino acid parallel strands of β-sheets crossing all its structure, passing through domains I, II, and III, the alternative to use ETX as a recombinant vaccine antigen consists on making mutations that reduce its toxicity, such as those in H106, Y196, or F199 (middle part). Moreover, fusing its whole sequence to the *C*-terminal part of other antigens, such as CPB, or expressing it on the cell surface of probiotic bacteria, such as *Lactobacillus casei*, are also interesting strategies (right part).

The data presented in Table 4 indicates that rETX with H106P mutation (rETX^H106P^) is the best-characterized vaccine candidate for protection against the animal diseases caused by *C. perfringens*. rETX^H106P^ is completely non-toxic and has been validated as a safe vaccine antigen against enterotoxemia [80,92,95,97]. Despite having low or null toxicity, rETX with V56C/F118C, S156E, and Y71A mutations have not been validated as potential vaccine antigens [82,85,100]. Other rETX, with Y196E-C and F199E mutations, have shown low toxicity and were used to vaccinate mice. Mice vaccinated with 15 µg of rETX^Y196E^-C were protected against challenge with 500 × LD_50_, although the toxicity of higher doses in farm animals is yet to be studied (Figure 3) [96].

Recently, Alimolaei et al. [97] produced an *L. casei* that presented an rETX^H106P^ antigen on its surface (LC-pT1NX-rETX^H106P^). Mice vaccinated orally with LC-pT1NX-rETX^H106P^ produced mucosal, humoral, and cellular immune responses, surviving the challenge with 200 × LD_50_ ETX. This strategy is promising and offers distinct advantages over parenteral routes due to the ease with which it can be orally administrated and the fact that it is safe because *L. casei* is not pathogenic. ETX^H106P^ is a well-known non-toxic mutant and, in this case, it also has the benefit of being carried by *L. casei*, an organism that is known to have probiotic effects that facilitate the development of mucosal immunity and other benefits for the immune system [97]. This strategy also proved to be effective in immune prophylaxis against tetanus [101]. Chandran et al. [94] used different amounts of formaldehyde-inactivated rETX per dose (50, 100, 200, 300, and 500 µg), as well as 200 µg of rETX co-administered with attenuated Sheep Pox Virus (SPV). The use of 50 µg and 100 µg per dose, induced antitoxin ETX titers of 3 and 5 IU/mL, respectively. There was no difference in the levels of antibodies generated by doses of 200, 300, and 500 µg, all of which produced approximately 7 IU/mL. This study demonstrated that the combination of recombinant and conventional vaccines is possible.

Similarly to the rETX used by Hunter et al. [90], Souza et al. [98] used rETX as a vaccine antigen and, hence, the protoxin (rPETX) was expressed without the sequence MKKNLVKSL at the *N*-terminal extremity. They transformed *E. coli* BL21 (DE3) strain with pET11a vector, resulting in rPETX in the form of inclusion bodies. Rabbits vaccinated with 50, 100, and 200 µg of formaldehyde-inactivated rPETX showed titers with 10, 30, and 40 IU/mL, respectively. The prime immunization was performed with rPETX associated with Freund’s complete adjuvant (FCA) and boosted with Freund's incomplete adjuvant (FIA). Moreira et al. [69] synthesized a CDS of ETX without the sequence for the 45 amino acids at the *N*-terminal portion using optimized codons for expression in *E. coli* and obtained a soluble rPETX that was non-toxic for MDCK cells. Rabbits vaccinated with 200 µg of rCPA, rCPB, and rPETX co-administered with Al(OH)_3_ as adjuvant showed titers of 25 IU/mL of antitoxin ETX. The dose of 200 µg of rETX used in the above studies induced different antitoxins titers, which may have been influenced by the various adjuvants used or by the presentation form of rETX (soluble or insoluble). It is important to bare in mind the fact that inclusion bodies also have adjuvant properties.

The rETXCPB chimera was developed, and its immunogenicity in mice was determined in a study by Langroudi, Shansara, and Aghaiypour [71]. This study was unclear as to the concentration of the protein used, and no adjuvant was employed. rETX was expressed in soluble form. Of specific interest was the direct use of CLS of *E. coli* expressing rETXCPB for the immunization of the animals. Even the antigens being used without any adjuvant were efficient in inducing levels of the ETX (6 IU/mL) and CPB (10 IU/mL) antitoxins. Thus, the use of both inclusion bodies and CLS has been proven to represent an alternative to conventional toxoids and purified recombinant antigens.

*E. coli* BL21 (DE3) is the most used system for the expression of rETX, rCPA, and rCPB. The strains of *E. coli* BL21 (DE3) pLysS™, Nova Blue (DE3) pLysS™, and Rosetta™ are the most commonly used, employing the pET22b and pET11a vectors. Chandran et al. [94] and Goswami et al. [102] used *E. coli* M15 and the pQE32 vector. This vector has an ampicillin resistance gene, and the expression of the target gene was under the control of the T5 promoter. The T5 promoter, in contrast to the T7 promoter, does not require co-expression of the bacteriophage RNA polymerase because it is recognized by the *E. coli* RNA polymerase. Using this system, these authors obtained 12 to 20 mg/L of purified rETX. The rETX obtained in *E. coli* M15 and BL21 (DE3) was expressed in the form of inclusion bodies. However, most of the other studies involving the expression of rETX obtained this antigen in a soluble form. Miyata et al. [103] demonstrated that removing the *C*-terminal portion (K274-K296) of ETX affects its solubility, which did not occur with the intact protoxin variants (rPETX) without the *N*-terminal extremity, suggesting that the amino acids present in the *C*-terminal region are essential for maintaining the stability and correct conformation of the ETX, and consequently the solubility.

## 5. Immunogenicity rCPA, rCPB, and rETX in Farm Animals

Numerous studies have described the use of recombinant toxins in illness immunoprophylaxis caused by *C. perfringens* in food animals (Table 5) [22,26,68,69,94,104]. Jiang et al. [105] evaluated the treatment of calves using a trivalent vaccine that contained 300 µg of each antigen (rCPA^(247–370)^, rCPB and rETX) emulsified in oil adjuvant ISA 15A VG. They obtained titers of 23.04, 33.7, and 9.43 IU/mL of antitoxin CPA, CPB, and ETX, respectively. However, this result should be interpreted with caution because the levels of antibodies were determined by ELISA, which measures total antibodies (neutralizing and non-neutralizing). On the other hand, Moreira et al. [69] evaluated the use of a trivalent vaccine containing 200 µg of each purified recombinant antigen (rCPA, rCPB, and rETX) adsorbed into adjuvant Al(OH)_3_ in ruminants. The titers of the CPA, CPB, and ETX antitoxins in cattle were 5.19, 13.71, and 12.74 IU/mL, respectively; 4.34, 13.71, and 7.66 IU/mL in sheep; and 4.70, 13.71, and 8.91 IU/mL in goats. Moreover, it should be noted that none of the recombinant antigens were inactivated with formaldehyde.

Despite the high potential of recombinant antigens in the immunoprophylaxis of animal diseases caused by *C. perfringens*, it is important to highlight two negative characteristics: (1) the production process and individual purification of each antigen can mean that this technology is not suitable for use in the veterinary industry; and (2) the fact that these antigens have portions that are not relevant to the generation of protective immune response (neutralizing antibodies). Thus, the development of recombinant chimeras containing two or more antigens has been spotlighted, either in immunoprophylaxis of clostridial, or other diseases [106,107,108]. The work of Zeng et al. [26] evaluated the capacity of two vaccine formulations, rCPA plus rCPB2B1 and a trivalent chimera rCPAB2B1, to generate neutralizing antitoxin in the serum and colostrum of swine and bovine. The individual titers of each antitoxin (CPA, CPB, and CPB2) were determined using culture supernatant of *C. perfringens* toxinotype C for the titration of antitoxin levels. An rCPA plus rCPB2B1 formulation induced titers of antitoxin 3 and 8 IU/mL in serum, and 1 and 6 IU/mL in swine and cow colostrum, respectively. The trivalent chimera rCPAB2B1 induced serum titers of 2 and 6 IU/mL, and 1 and 2 IU/mL in the colostrum of sows and cows respectively. Similarly, as per the studies on mice, the trivalent chimera rCPAB2B1 induced lower titers both in serum and in the colostrum of swine and beef matrices.

The recombinant chimeras in the above-cited studies eliminate the need for production of the individual antigens. However, these chimeras were constructed using intact toxins. Furthermore, the low titers of antitoxin generated by trivalent chimera rCPAB2B1 suggest a possible inappropriate conformation of the molecule, masking, or changing protective epitopes. Thus, the identification of the protective areas of each toxin provides important data for the construction of multivalent recombinant chimeras. Work involving the use of protective domains of toxins from *C. perfringens* and other pathogens has been performed [41,43,97]. The construction of a chimera composed of CPA-C^(247–370)^, CPB-C^(143–311)^, and ETX^H106P^ may be a viable and promising method of immunoprophylaxis against the animal illness caused by *C. perfringens*.

When the production of veterinary vaccines involves the use of purified antigen, the expression of insoluble proteins involves a laborious and time-consuming production process, since solubilization steps, refolding, and purification are required. Moreover, these processes encumber the manufacturing process, which makes recombinant antigens less commercially competitive than conventional toxoids. An excellent alternative has been demonstrated through the immunization of animals with non-purified recombinant antigens, which consist in the use of cellular fractions (inclusion bodies or supernatant lysate) containing the recombinant antigen, obtained after cell lysis. Lobato et al. [22] used ~200 µg of inclusion bodies of rETX inactivated with formaldehyde directly to vaccinate animals and obtained titers of 40, 14.3, 26, and 13.1 IU/mL of neutralizing antitoxins in rabbits, goats, sheep, and cattle, respectively. Zeng et al. [26] also demonstrated the potential of rCPA inclusion bodies and polyvalent chimeras in the vaccination of mice, cattle, and sows. Inclusion bodies can be easily obtained from cell lysis and semi-purified by centrifuging and washing cycles. In addition, increasing the stability of the antigen, interfering with the action of proteases, and acting as an immunological adjuvant by deposit effect slowly releases the antigen and delays the recognition by the immune system.

The elimination of solubilization and refolding steps, as well as the purification of recombinant antigens, represented a significant advancement. However, a simpler strategy for the production of recombinant vaccines against *C. botulinum* was proposed by Moreira Jr et al. [27]. Vaccines against botulinum toxin serotypes C and D were evaluated in three ways: (1) purified recombinant antigens; (2) recombinant *E. coli* bacterin; and (3) cell lysate fraction (inclusion bodies + supernatant lysis). The purified antigens induced in guinea pigs were 13 and 21 IU/mL of antitoxin C and D respectively. Interestingly, recombinant bacterins and cell lysate factions induced titers of 12 IU/mL of anti-C and 20 IU/mL of anti-D. The use of unpurified recombinant antigens appears as a simple alternative, reducing the time and cost involved in the process by which recombinant veterinary vaccines are produced while also maintaining the quality of the antigens used. Therefore, a recombinant chimera containing protector domains that does not require purification would represent a promising vaccine against *C. perfringens* toxins. A problem pointed out in the literature for production of recombinant antigens in *E. coli* is potential endotoxins (LPS) contamination, which are pyrogenic to humans and animals [109]. However, there were no side-effects reported when unpurified antigens were used in the immunization of guinea pigs [27].

The revaccination of animals vaccinated with conventional toxoid is recommended every six months. Therefore, in addition to the requirement for a simplified production process, the choice of adjuvant for the prolongation of the immune response is extremely important. The use of Al(OH)_3_ as an adjuvant is common in clostridial recombinant vaccines. However, Al(OH)_3_ and saponins induce immune responses that are short in duration; hence, revaccination is recommended two or more times a year [110]. Oil adjuvants induce higher and lasting antibody titers. As such, they are only suitable for use in annual vaccinations, which is desirable for veterinary vaccines [111,112]. Consequently, studies related to the determination of the dynamics of antibodies in animals vaccinated with recombinant antigenic domains, mono- or polyvalent (chimeras or co-administered), associated with different adjuvants, are necessary to determine the immunogenicity of these formulations in production animals.

In terms of antigen dose, the work performed by Chandran et al. [94], evaluated the doses of 50, 100, 200, 300, and 500 μg of rETX in sheep, where doses of 50 and 100 μg generated titers less than 5 IU/mL, and other doses showed no difference in the induction of immune response, generating 7 IU/mL. The subcutaneous was the main route of inoculation in the evaluation of the recombinant toxins; however, some species, such as horses, exhibited exacerbated reactions when vaccinated by this route. The intervals between the first dose and booster ranged from 7 to 35 days between works. The antigen concentration, route of administration, and the dosing interval are factors that also influence the duration of the immune response; therefore, a detailed assessment of the role these factors play in the immunization of each particular specie is of importance.

Some species, such as goats, demonstrate titers of neutralizing antibody of short duration, demanding revaccination every three to four months [113]. Bernáth et al. [114] found that eight weeks between vaccinations is required for toxoid-vaccinated sheep when the Al(OH)_3_ adjuvant is employed. The first dose provides immunity until the administration of the second dose. After that, a second peak in the antibodies is achieved, providing protection for a prolonged period. This work only accompanied the titers during three months [114]. It would be ideal to monitor the dynamics of the antibodies for a year, or during the full period of which the serum antitoxins are detected. These data were obtained from vaccinations with toxoids of *Clostridium* spp., which have well-known problems in inducing titers of neutralizing antibodies due to variability in batches, excessive antigens, and the utilization of formaldehyde to inactivate the toxins. Therefore, the evaluation of all the above factors, such as an adjuvant, administration route, dosing interval, and antigen concentration in different species is essential to facilitate the development and use of veterinary vaccines that contain rCPA, rCPB, and rETX.

## 6. Conclusions

Recombinant toxins from *C. perfringens* have been efficient on inducing protective immune response in farm animals. Studies over the characteristics of the both CPA and CPB defined that only their *C*-terminal parts can confer protection. Due to structural reasons, this is not true for ETX, to which the development of non-toxic mutants of the whole protein is the main strategy for a vaccine antigen. With these data, it seems that the next step is the construction of a chimeric antigen containing the *C*-terminal parts of CPA and CPB, together with a mutated ETX. Furthermore, the use of fused antigens appears to be interesting for vaccine design, since many published studies are working on this strategy.

Although most of the effective experimental vaccines have been successfully produced in laboratory scale with purified proteins, the same procedure is not attractive for the veterinary vaccine industry. The purification step, for example, increases production expenses and, thus, can impair the use of recombinant antigens in animal vaccines instead of the conventional toxoids. To solve this drawback, the use of SLC, inclusion bodies, or even recombinant bacterins have shown to be a reasonable and low-cost alternative for large-scale production.

## Figures and Tables

**Table 1:** Main diseases caused by *Clostridium perfringens* toxinotypes in production animals.

Toxinotype	Produced Toxins	Diseases (Affected Animals)
A	CPA	Gas gangrene (all production species) and enterotoxemia (ovine)
	CPA, CPE	Enteritis (equine, caprine, and swine)
	CPA, NetB	Necroticenteritis (poultry)
	CPA, NetF	Neonatal necroticenteritis (foal)
	CPA, CPB2	Necrotic enteritis (piglets), abomasitis (calves), enterocolitis (foal)
B	CPA, CPB, ETX	Necrotic enteritis and hemorrhagic enterotoxemia (bovine, ovine, and equine)
C	CPA, CPB	Necrotic enteritis and enterotoxemia (bovine, ovine, caprine, swine, and newborn equine); necrotic enteritis (poultry)
D	CPA, ETX	Enterotoxemia (ovine, bovine, and caprine)
E	CPA, CPI	Hemorrhagic enteritis (lambs, and calves)

Adapted from Revitt-Mill, Rood, and Adans [4] and Li et al. [11].

**Table 2:** Immunogenicity of rCPA in model animals.

Molecule	Doses	Via	No. of Doses	Interval (Days)	Adjuvant	Animal Model	Challenge	Survival (%)	References
rGST-CPA-C^(247−370)^	10 µg	IP	3	14	FIA	Mouse	1 µg of CPA; CAA	100 (6/6)	[46]
							25 µg Cpb	83.3 (5/6)	
rCPA-N^(1–249)^ ^A^ rCPA-C^(247^^–370)^ ^B^ rGST-CPA-C^(247^^–370)^ ^C^	0.36 pM	IP	2–6	14	FIA	Mouse	50 × MLD CPA	0 (0/6) ^A^	[44]
								100 (6/6) ^C^	
								100 (6/6) ^B,C^	
							10^9^ CFU (10 × LD_100_)	0 (0/6) ^A^	
								100 (6/6) ^B,C^	
								66.6 (4/6) ^C^	
rGST-CPA-C	0.36 pM	IP	2–6	14	FIA	Mouse	15 µg CPA	100 (6/6)	[47]
rCPA-C^(251–370)^	10 µg	IP	2	14	FCA/FIA	Mouse	1 µg or 10^8^ CFU	100 (10/10)	[48]
rCPA-C^(281–370)^								100 (10/10)	
rCPA-C^(311–370)^								60 (6/10)	
rGST-CPA-C^(247–370)^	10^9^ CFU (75 ng)	IP	3	14	None	Mouse	12 × LD_50_	NE	[40]
*B. subtilis*	5 × 10^10^ (3.6 µg)	PO		21				100 (6/6)	
(Cell surface display)	2 × 10^9^ (150 ng)	IN		21				100 (6/6)	
rCPAE (CPA-C^(284–398)^ + CPE-C^(^^197–312)^)	30 µg	SC	4	7–14	FCA/FIA	Mouse	5 × LD_50_ CPA	100 (12/12)	[43]
							5 × LD_50_ CPE	75 (9/12)	
rCS (CPA-C^(284–398)^ + SAA^(36–221)^)	50 µg	SC/IP	3	14	FCA/FIA	Mouse	5 × LD_100_ CPA	100 (6/6)	[41,42]
							5 × LD_100_ SAA	100 (6/6)	
							5 × LD_100_ CPA + SAA	83.3 (5/6)	
rCPA	100 µg	SC	2	14	Al(OH)_3_	Mouse	1 × LD_100_^A^ 2 × LD_100_^B^ 1 × LD_100_^C^	80 ^A^, 70 ^B^, 83 ^C^	[26]
rCPB2B1								90 ^A^, 73 ^B^, 93 ^C^	
rCPA + CPB2B1								100 ^A,B,C^	
rCPAB2B1								93 ^B^, 100 ^A,C^	

rCPA: recombinant *Clostridium perfringen*s Alpha toxin; rCPA-N: recombinant *C. perfringen*s Alpha toxin *N*-terminal domain; rCPA-C: recombinant *C. perfringen*s Alpha toxin *C*-terminal domain; rGST-CPA-C: recombinant *C. perfringen*s Alpha toxin *C*-terminal domain fused with Glutathion S-transferase (GST); CPE: *C. perfringens* Enterotoxin; SAA: *Staphylococcus aureus* Alpha-hemolysin; rCPB2B1: recombinant *C. perfringens* Beta and Beta 2 toxins fused; rCPAB2B1: recombinant *C. perfringens* Alpha, Beta and Beta 2 toxins fused; IP: intraperitoneal; PO: oral administration (*per os*); IN: Intranasal; SC: Subcutaneous; FCA: Freund’s complete adjuvant; FIA: Freund’s incomplete adjuvant; CAA: *Clostridium absonum* Alpha toxin; Cpb: *Clostridium bifermentans* phospholipase C; MLD: Mouse lethal dose; CFU: Colony-forming unit; NE: Not evaluated; ^A,B,C^ The indicated survival percentage corresponds to either the antigens in the first column, or the challenge, which are marked with the same letter.

**Table 3:** Immunogenicity of rCPB in model animals.

Molecule	Doses	Via	No. of Doses	Interval (Days)	Adjuvant	Animal Model	Challenge	Protection (IU/mL) or Survival (%)	References
rCPB	100 µg	SC	2	21	Al(OH)_3_	Rabbit	-	10 IU/mL	[67]
rETXCPB (ETX ^A^ + CPB ^B^)	0.5 mL	IP	2	21	None	Rabbit	-	6 ^A^ and 10 ^B^ IU/mL	[71]
rCPA	200 µg	SC	2	21	Al(OH)_3_	Rabbit	-	9.6 IU/mL	[68]
rCPB								20.4 IU/mL	
rCPIB	30 µg	SC	3	14	FCA/FIA	Mouse	5 × LD_100_ CPB	83% (10/12)	[70]
(CPI-C^(466–665)^ + CPB-C^(143–311)^)							5 × LD_100_ CPI	91% (11/12)	

CPB: *Clostridium perfringens* Beta toxin; ETX: *C. perfringens* Epsilon toxin; CPI: *C. perfringens* Iota toxin; rCPB: recombinant *C. perfringens* Beta toxin; rCPA: recombinant *C. perfringens* Alpha toxin; CPI-C: *C. perfringens* Iota toxin *C*-terminal domain; CPB-C: *C. perfringens* Beta toxin *C*-terminal domain; SC: Subcutaneous; IP: Intraperitoneal; FCA: Freund’s complete adjuvant; FIA: Freund’s incomplete adjuvant. ^A,B^ The indicated survival percentage corresponds to the antigens in the first column marked with the same letter.

**Table 4:** Immunogenicity of rETX in model animals.

Molecule	Doses	Via	No. of Doses	Interval (Days)	Adjuvant	Animal Model	Challenge	Survival (%) or Protection (IU/mL)	References
rETX	50; 100; 200; 300; 500 µg	SC	2	21	Al(OH)_3_	Rabbit	-	3; 5; 7; 7; 8; 7 IU/mL	[94]
rETX	50; 100; 200 µg	SC	5	2–10	FCA/FIA	Rabbit	-	10; 30; 40 IU/mL	[98]
rCPA, rCPB, rETX	200 µg	SC	2	21	Al(OH)_3_	Rabbit	-	9.6; 24.4; 25 IU/mL	[69]
rETX^H106P^	0.27 nmol	IP	3	14–21	FIA	Mouse	100 and 1000 × LD_50_	100% (30/30)	[80]
rETX^H106P^	10 µg	SC	3	17–21	Al(OH)_3_	Mouse	100 × LD_50_	100% (3/3)	[95]
rETX^F199E^									
rETX^Y196E^-C	5 µg ^A^	SC IP	3	14	FCA	Mouse	100 × LD_50_^a^	100% (5/5) ^Aab,Bab,Cab^	[96]
	10 µg ^B^						500 × LD_50_^b^	20 ^Cb^; 80 ^Aa^; 100% ^Ba,Ca^ (1; 4; 5/5)	
	15 µg ^C^						1000 × LD_50_^c^	80% (4/5) ^Ca^	
*Lactobacillus casei*	10^9^ CFU	IG	3	16–21	None	Mouse	200 × LD_50_	100% (10/10)	[97]
(Cell surface display)									
rETX^H106P^									
rETXCPB (ETX ^A^ + CPB ^B^)	0.5 mL	IP	2	21	None	Rabbit	-	6 ^A^ and 10 ^B^ IU/mL	[71]

CPB: *Clostridium perfringens* Beta toxin; ETX: *C. perfringens* Epsilon toxin; rCPA: recombinant *C. perfringen*s Alpha toxin; rETX: recombinant *C. perfringens* Epsilon toxin; rCPA: recombinant *C. perfringens* Alpha toxin; rCPB: recombinant *C. perfringens* Beta toxin; rETX^H106P^: recombinant *C. perfringens* Epsilon toxin with mutation in H106 amino acid; rETX^F199E^: recombinant *C. perfringens* Epsilon toxin with mutation in F199 amino acid; rETX^Y196E^-C: recombinant *C. perfringens* Epsilon toxin with mutation in Y196 amino acid and containing *C*-terminal domain; CFU: Colony-forming unit; SC: Subcutaneous; IP: Intraperitoneal; IG: Intragastric; FCA: Freund’s complete adjuvant; FIA: Freund’s incomplete adjuvant. ^a,b,c,A,B,C^ The indicated survival percentage corresponds to either the antigens in the first column, or the challenge, which are marked with the same letter.

**Table 5:** Immune response against CPA, CPB, and ETX generated by recombinant antigens in farm animals.

Antigens	Doses (via)	Boost Dose (Day)	Adjuvant	Animal	Method	Protection (UI/mL)				References
rETX	50, 100, 200, 300, and 500 μg (SC)	35	Al(OH)_3_	Sheep	SN	2; 5; 7; 7; 9; 9				[94]
rETX	200 μg (SC)	14	Al(OH)_3_	Cattle	SN	13.1				[22]
				Sheep		26				
				Goat		14.3				
rCPA rCPB2B1 rCPA+rCPB2B2 rCPAB2B1	200 μg (SC)	14	Al(OH)_3_ gel	**Animal/specimen**	SN	**rCPA**	**rCPB2B1**	**rCPAB2B1**	**rCPA + rCB2B1**	[26]
				Cattle/Serum		1	2	2	3	
				Cattle/Colostrum		0	1	1	1	
				Swine/Serum		4	6	6	6	
				Swine/Colostrum		1	2	2	8	
rCPA, rCPB, rETX	300 μg (SC)	14	ISA 15A VG	Calves	ELISA	**CPA**	**CPB**		**ETX**	[105]
						23.04	33.7		9.43	
rCPA, rCPB	200 μg (SC)	35	Al(OH)_3_	Swine	SN	6	14.5		-	[68]
				Piglets		4.2	10.9		-	
rCPA, rCPB, rETX	200 μg (SC)	35	Al(OH)_3_	Cattle	SN	5.19	13.71		12.74	[69]
				Sheep		4.34	13.71		7.66	
				Goat		4.7	13.71		8.91	

CPA: *Clostridium perfringens* Alpha toxin; CPB: *C. perfringens* Beta toxin; ETX: *C. perfringens* Epsilon toxin; rCPA: recombinant *C. perfringen*s Alpha toxin; rCPB: recombinant *C. perfringens* Beta toxin; rETX: recombinant *C. perfringens* Epsilon toxin; rCPB2B1: recombinant *C. perfringens* Beta and Beta 2 toxins fused; rCPAB2B1: recombinant *C. perfringens* Alpha, Beta and Beta 2 toxins fused; SC: Subcutaneous; SN: Serum neutralization assay.

## References

[1] McClane B.A., Uzal F.A., Miyakawa M., Lierly D., Wilkins T.D., Dworkin M., Falkow S., Rosenburg E., Schleifer K.H., Stackebrandt E. (2006). The enterotoxic clostridia. The Prokaryotes.

[2] Hatheway C.L. (1990). Toxigenic clostridia. Clin. Microbiol. Rev..

[3] McClane B.A., Robertson S., Li J., Doyle M.P., Buchanan R.L. (2013). *Clostridium* *perfringens*. Food Microbiology: Fundamentals and Frontiers.

[4] Revitt-Mills S., Rood J., Adams V. (2015). *Clostridium perfringens* extracellular toxins and enzymes: 20 and counting. Microbiol. Aust..

[5] Nillo L. (1980). *Clostridium perfringens* in Animal Disease: A Review of Current Knowledge. Can. Vet. J..

[6] Uzal F.A., Vidal J.E., McClane B.A., Gurjar A.A. (2010). *Clostridium perfringens* toxins involved in mammalian veterinary diseases. Changes.

[7] Songer J.G. (1996). Clostridial enteric diseases of domestic animals. Clin. Microbiol. Rev..

[8] Titball R.W., Rood J.I. (2002). *Clostridium perfringens*: Wound infections. Molecular Medical Microbiology.

[9] Manteca C., Daube G., Jauniaux T., Linden A., Pirson V., Detilleux J., Ginter A., Coppe P., Kaeckenbeeck A., Mainil J. (2002). A role for the *Clostridium perfringens* β2 toxin in bovine enterotoxaemia?. Vet. Microbiol..

[10] Dutra I. Clostridioses—Doenças Que Mais Matam Bovinos. http://www.beefpoint.combr/radares-tecnicos/sanidade/clostridioses-doencas-que-mais-matam-bovinos-5068/.

[11] Li J., Adams V., Bannam T.L., Miyamoto K., Garcia J.P., Uzal F.A., Rood J.I., McClane B.A. (2013). Toxin plasmids of *Clostridium perfringens*. Microbiol. Mol. Biol. Rev..

[12] Lebrun M., Mainil J.G., Linden A. (2010). Cattle enterotoxaemia and *Clostridium perfringens*: Description, diagnosis and prophylaxis. Vet. Rec..

[13] Massey P.R., Sakran J.V., Mills A.M., Sarani B., Aufhauser D.D., Sims C.A., Pascual J.L., Kelz R.R., Holena D.N. (2012). Association for Academic Surgery Hyperbaric oxygen therapy in necrotizing soft tissue infections. J. Surg. Res..

[14] Garcia J.P., Beingesser J., Bohorov O., Bohorova N., Goodman C., Kim D., Pauly M., Velasco J., Whaley K., Zeitlin L. (2014). Prevention and treatment of *Clostridium perfringens* epsilon toxin intoxication in mice with a neutralizing monoclonal antibody (c4D7) produced in *Nicotiana benthamiana*. Toxicon.

[15] Wong C.H.H., Chang H.C.C., Pasupathy S., Khin L.W.W., Tan J.L.L., Low C.O.O. (2003). Necrotizing fasciitis: Clinical presentation, microbiology, and determinants of mortality. J. Bone Jt. Surg. Am..

[16] Lobato F.C.F., Moro E., Umehara O. (2000). Avaliação da resposta de antitoxinas beta e épsilon de *Clostridium perfringens* induzidas em bovinos e coelhos por seis vacinas camerciais no Brasil. Arq. Bras. Med. Vet. Zootec..

[17] Veschi J., Dutra I., Aalves M., Perri S., Zafalon L., Fernandez-Miyakawa M. (2012). Sorological evaluation of polyvalent commercial vaccines against enterotoxemia in goats. ARS Vet..

[18] Bhatia B., Solanki A.K., Kaushik H., Dixit A., Garg L.C. (2014). B-cell epitope of beta toxin of *Clostridium perfringens* genetically conjugated to a carrier protein: Expression, purification and characterization of the chimeric protein. Protein Expr. Purif..

[19] Byrne M.P., Smith L.A. (2000). Development of vaccines for prevention of botulism. Biochimie.

[20] Bokori-Brown M., Hall C., Vance C., Costa S.F., Savva C.G., Naylor C.E., Cole A.R., Basak A.K., Moss D.S., Titball R.W. (2014). *Clostridium perfringens* epsilon toxin mutant Y30A-Y196A as a recombinant vaccine candidate against enterotoxemia. Vaccine.

[21] Thaysen-Andersen M., Jørgensen S.B., Wilhelmsen E.S., Petersen J.W., Højrup P. (2007). Investigation of the detoxification mechanism of formaldehyde-treated tetanus toxin. Vaccine.

[22] Lobato F.C.F., Lima C.G.R.D., Assis R.A., Pires P.S., Silva R.O.S., Salvarani F.M., Carmo A.O., Contigli C., Kalapothakis E. (2010). Potency against enterotoxemia of a recombinant *Clostridium perfringens* type D epsilon toxoid in ruminants. Vaccine.

[23] Cavalcanti M., Porto T., Porto A., Brandi I., Lima Filho J., Pessoa Junior A. (2004). Large scale purification of *Clostridium perfringens* toxins: A review. Braz. J. Pharm. Sci..

[24] Larentis A., Nicolau J.F.M.Q., Esteves G.D., Vareschini D., de Almeida F.V., dos Reis M., Galler R., Medeiros M. (2014). Evaluation of pre-induction temperature, cell growth at induction and IPTG concentration on the expression of a leptospiral protein in *E. coli* using shaking flasks and microbioreactor. BMC Res. Notes.

[25] Moreira G., Cunha C., Salvarani F., Gonçalves L., Pires P., Conceição F., Lobato F. (2014). Production of recombinant botulism antigens: A review of expression systems. Anaerobe.

[26] Zeng J., Deng G., Wang J., Zhou J., Liu X., Xie Q., Wang Y. (2011). Potential protective immunogenicity of recombinant clostridium perfringens α-β2-β1 fusion toxin in mice, sows and cows. Vaccine.

[27] Moreira C., Cunha C.E.P., Moreira G.S.M.G., Mendonça M., Salvarani F.M., Moreira A.N., Conceição F.R. (2016). Protective potential of recombinant non-purified botulinum neurotoxin serotypes C and D. Anaerobe.

[28] Titball R.W., Naylor C.E., Basak A.K. (1999). The *Clostridium perfringens* alpha-toxin. Anaerobe.

[29] Flores-Díaz M., Alape-Girón A. (2003). Role of *Clostridium perfringens* phospholipase C in the pathogenesis of gas gangrene. Toxicon.

[30] Sakurai J., Nagahama M., Oda M. (2004). *Clostridium perfringens* alpha-toxin: Characterization and mode of action. J. Biochem..

[31] Oda M., Terao Y., Sakurai J., Nagahama M. (2015). Membrane-binding mechanism of *Clostridium perfringens* alpha-toxin. Toxins.

[32] Nagahama M., Okagawa Y., Nakayama T., Nishioka E., Sakurai J. (1995). Site-directed mutagenesis of histidine residues in *Clostridium perfringens* alpha-toxin. J. Bacteriol..

[33] Nagahama M., Nakayama T., Michiue K., Sakurai J. (1997). Site-specific mutagenesis of *Clostridium perfringens* alpha-toxin: Replacement of Asp-56, Asp-130, or Glu-152 causes loss of enzymatic and hemolytic activities. Infect. Immun..

[34] Takehara M., Takagishi T., Seike S., Ohtani K., Kobayashi K., Miyamoto K., Shimizu T., Nagahama M. (2016). *Clostridium perfringens* α-toxin impairs innate immunity via inhibition of neutrophil differentiation. Sci. Rep..

[35] Titball R.W., Hunter S.E., Martin K.L., Morris B.C., Shuttleworth A.D., Rubidge T., Anderson D.W., Kelly D.C. (1989). Molecular cloning and nucleotide sequence of the alpha-toxin (phospholipase C) of *Clostridium perfringens*. Infect. Immun..

[36] Leslie D., Fairweather N., Pickard D., Dougan G., Kehoe M. (1989). Phospholipase C and haemolytic activities of *Clostridium perfringens* alpha-toxin cloned in *Escherichia coli*: Sequence and homology with a *Bacillus cereus* phospholipase C. Mol. Microbiol..

[37] Okabe A., Tohru S., Hayashi H. (1989). Cloning and sequencing of a phospholipase C gene of *Clostrdium perfringens*. Biochem. Biophys. Res. Commun..

[38] Saint-Joanis B., Garnier T., Cole S. (1989). Gene cloning shows the alpha-toxin of *Clostridium perfringens* to contain both sphingomyelinase and lecithinase activities. Mol. Gen. Genet..

[39] Tso J., Siebel C. (1989). Cloning and expression of the phospholipase C gene from *Clostridium perfringens* and *Clostridium bifermentans*. Infect. Immun..

[40] Hoang T.H., Hong H.A., Clark G.C., Titball R.W., Cutting S.M. (2008). Recombinant *Bacillus subtilis* expressing the *Clostridium perfringens* alpha toxoid is a candidate orally delivered vaccine against necrotic enteritis. Infect. Immun..

[41] Uppalapati S.R., Kingston J.J., Murali H.S., Batra H.V. (2014). Heterologous protection against alpha toxins of *Clostridium perfringens* and *Staphylococcus aureus* induced by binding domain recombinant chimeric protein. Vaccine.

[42] Uppalapati S.R., Kingston J.J., Murali H.S., Batra H.V. (2012). Generation and characterization of an inter-generic bivalent alpha domain fusion protein αCS from *Clostridium perfringens* and *Staphylococcus aureus* for concurrent diagnosis and therapeutic applications. J. Appl. Microbiol..

[43] Shreya D., Uppalapati S.R., Kingston J.J., Sripathy M.H., Batra H.V. (2015). Immunization with recombinant bivalent chimera r-Cpae confers protection against alpha toxin and enterotoxin of *Clostridium perfringens* type A in murine model. Mol. Immunol..

[44] Williamson E.D., Titball R.W. (1993). A genetically engineered vaccine against the alpha-toxin of *Clostridium perfringens* protects mice against experimental gas gangrene. Vaccine.

[45] Schoepe H., Pache C., Neubauer A., Potschka H., Schlapp T., Wieler L.H., Baljer G. (2001). Naturally occurring *Clostridium perfringens* nontoxic alpha-toxin variant as a potential vaccine candidate against alpha-toxin-associated diseases. Infect. Immun..

[46] Neeson B.N., Clark G.C., Atkins H.S., Lingard B., Titball R.W. (2007). Analysis of protection afforded by a *Clostridium perfringens* α-toxoid against heterologous clostridial phospholipases C. Microb. Pathog..

[47] Ginter A., Williamson E.D., Dessy F., Coppe P., Bullifent H., Howells A., Titball R.W. (1996). Molecular variation between the alpha-toxins from the type strain (NCTC 8237) and clinical isolates of *Clostridium perfringens* associated with disease in man and animals. Microbiology.

[48] Nagahama M., Oda M., Kobayashi K., Ochi S., Takagishi T., Shibutani M., Sakurai J. (2013). A recombinant carboxy-terminal domain of alpha-toxin protects mice against *Clostridium perfringens*. Microbiol. Immunol..

[49] Guillouard I., Garnier T., Cole S.T. (1996). Use of site-directed mutagenesis to probe structure-function relationships of alpha-toxin from *Clostridium perfringens*. Infect. Immun..

[50] Guillouard I., Alzari P.M., Saliou B., Cole S.T. (1997). The carboxy-terminal C2-like domain of the alpha-toxin from *Clostridium perfringens* mediates calcium-dependent membrane recognition. Mol. Microbiol..

[51] Alape-Girón A., Flores-Díaz M., Guillouard I., Naylor C., Titball R.W., Rucavado A., Lomonte B., Basak A., Gutiérrez J., Cole S. (2000). Identification of residues critical for toxicity in *Clostridium perfringens* phospholipase C, the key toxin in gas gangrene. Eur. J. Biochem..

[52] Schoepe H., Neubauer A., Schlapp T., Wieler L.H., Baljer G. (2006). Immunization with an alphatoxin variant 121A/91-R212H protects mice against *Clostridium perfringens* alphatoxin. Anaerobe.

[53] Goossens E., Verherstraeten S., Valgaeren B.R., Pardon B., Timbermont L., Schauvliege S., Rodrigo-Mocholí D., Haesebrouck F., Ducatelle R., Deprez P.R. (2016). The *C*-terminal domain of *Clostridium perfringens* alpha toxin as a vaccine candidate against bovine necrohemorrhagic enteritis. Vet. Res..

[54] Titball R.W. (2005). Gas gangrene: An open and closed case. Microbiology.

[55] Kulkarni R.R., Parreira V.R., Sharif S., Prescott J.F. (2007). Immunization of broiler chickens against *Clostridium perfringens*-induced necrotic enteritis. Clin. Vaccine Immunol..

[56] Nuccitelli A., Cozzi R., Gourlay L.J., Donnarumma D., Necchi F., Norais N., Telford J.L., Rappuoli R., Bolognesi M., Maione D. (2011). Structure-based approach to rationally design a chimeric protein for an effective vaccine against Group B *Streptococcus* infections. Proc. Natl. Acad. Sci. USA.

[57] Gurjar A., Li J., Mcclane B.A. (2010). Characterization of toxin plasmids in *Clostridium perfringens* type C isolates. Infect. Immun..

[58] Nagahama M., Ochi S., Oda M., Miyamoto K., Takehara M., Kobayashi K. (2015). Recent insights into *Clostridium perfringens* beta-toxin. Toxins.

[59] Sakurai J., Nagahama M. (2006). *Clostridium perfringens* beta-toxin: Characterization and action. Toxin Rev..

[60] Hunter S.E.C., Brown J.E., Oyston P.C.F., Sakurai J., Titball R.W. (1993). Molecular genetic analysis of beta-toxin of *Clostridium perfringens* reveals sequence homology with alpha-toxin, gamma-toxin, and leukocidin of *Staphylococcus aureus*. Infect. Immun..

[61] Gurtner C., Popescu F., Wyder M., Sutter E., Zeeh F., Frey J., von Schubert C., Posthaus H. (2010). Rapid cytopathic effects of *Clostridium perfringens* beta-toxin on porcine endothelial cells. Infect. Immun..

[62] Popescu F., Wyder M., Gurtner C., Frey J., Cooke R.A., Greenhill A.R., Posthaus H. (2011). Susceptibility of primary human endothelial cells to *C. perfringens* beta-toxin suggesting similar pathogenesis in human and porcine necrotizing enteritis. Vet. Microbiol..

[63] Autheman D., Wyder M., Popoff M., D’Herde K., Christen S., Posthaus H. (2013). *Clostridium perfringens* beta-toxin induces necrostatin-inhibitable, calpain-dependent necrosis in primary porcine endothelial cells. PLoS ONE.

[64] Roos S., Wyder M., Candi A., Regenscheit N., Nathues C., van Immerseel F., Posthaus H. (2015). Binding Studies on isolated porcine small intestinal mucosa and in vitro toxicity studies reveal lack of effect of *C. perfringens* beta-toxin on the porcine intestinal epithelium. Toxins.

[65] Steinthorsdottir V., Fridriksdottir V., Gunnarsson E., Andrésson O.S. (1998). Site-directed mutagenesis of *Clostridium perfringens* beta-toxin: Expression of wild-type and mutant toxins in *Bacillus subtilis*. FEMS Microbiol. Lett..

[66] Steinthorsdottir V., Halldórsson H., Andrésson O.S. (2000). *Clostridium perfringens* beta-toxin forms multimeric transmembrane pores in human endothelial cells. Microb. Pathog..

[67] Milach A., de los Santos J.R., Turnes C.G., Moreira A.N., de Assis R.A., Salvarani F.M., Lobato F.C.F., Conceição F.R. (2012). Production and characterization of *Clostridium perfringens* recombinant β toxoid. Anaerobe.

[68] Salvarani F.M., Conceição F.R., Cunha C.E.P., Moreira G.M.S.G., Pires P.S., Silva R.O.S., Alves G.G., Lobato F.C.F. (2013). Vaccination with recombinant *Clostridium perfringens* toxoids α and β promotes elevated antepartum and passive humoral immunity in swine. Vaccine.

[69] Moreira G.M.S.G., Salvarani F.M., Cunha C.E.P., Mendonça M., Moreira A.N., Gonçalves L.A., Pires P.S., Lobato F.C.F., Conceição F.R. (2016). Immunogenicity of a trivalent recombinant vaccine against *Clostridium perfringens* alpha, beta, and epsilon toxins in farm ruminants. Sci. Rep..

[70] Shreya D., Majumder S., Kingston J.J., Batra H.V. (2016). Generation and characterization of recombinant bivalent fusion protein r-Cpib for immunotherapy against *Clostridium perfringens* beta and iota toxemia. Mol. Immunol..

[71] Langroudi R.P., Shamsara M., Aghaiypour K. (2013). Expression of *Clostridium perfringens* epsilon-beta fusion toxin gene in *E. coli* and its immunologic studies in mouse. Vaccine.

[72] Nagahama M., Hayashi S., Morimitsu S., Sakurai J. (2003). Biological activities and pore formation of *Clostridium perfringens* beta toxin in HL 60 cells. J. Biol. Chem..

[73] Bai J., Zhang Y., Zhao B. (2006). Cloning of alpha-beta fusion gene from *Clostridium perfringens* and its expression. World J. Gastroenterol..

[74] Bakhshi F., Langroudi R.P., Eimani B.G. (2011). Enhanced expression of recombinant beta toxin of *Clostridium perfringens* type B using a commercially available *Escherichia coli* strain. Onderstepoort J. Vet. Res..

[75] Alves G.G., Ávila R.A.M., Chávez-Olórtegui C.D., Lobato F.C.F. (2014). *Clostridium perfringens* epsilon toxin: The third most potent bacterial toxin known. Anaerobe.

[76] Minami J., Katayama S., Matsushita O., Matsushita C., Okabe A. (1997). Lambda-toxin of *Clostridium*
*perfringens* activates the precursor of epsilon-toxin by releasing its *N*- and *C*-terminal peptides. Microbiol. Immunol..

[77] Miyata S., Matsushita O., Minami J., Katayama S., Shimamoto S., Okabe A. (2001). Cleavage of a *C*-terminal peptide is essential for heptamerization of *Clostridium perfringens* ε-toxin in the synaptosomal membrane. J. Biol. Chem..

[78] Bokori-Brown M., Savva C.G., Costa S.P.F., Naylor C.E., Basak A.K., Titball R.W. (2011). Molecular basis of toxicity of *Clostridium perfringens* epsilon toxin. FEBS J..

[79] Freedman J.C., McClane B.A., Uzal F.A. (2016). New insights into *Clostridium perfringens* epsilon toxin activation and action on the brain during enterotoxemia. Anaerobe.

[80] Oyston P.C.F., Payne D.W., Havard H.L., Williamson E.D., Titball R.W. (1998). Production of a non-toxic site-directed mutant of *Clostridium perfringens* epsilon-toxin which induces protective immunity in mice. Microbiology.

[81] Ivie S.E., McClain M.S. (2012). Identification of amino acids important for binding of *Clostridium perfringens* epsilon toxin to host cells and to HAVCR1. Biochemistry.

[82] Jiang Z., Chang J., Wang F., Yu L. (2015). Identification of tyrosine 71 as a critical residue for the cytotoxic activity of *Clostridium perfringens* epsilon toxin towards MDCK cells. J. Microbiol..

[83] Ivie S.E., Fennessey C.M., Sheng J., Rubin D.H., McClain M.S. (2011). Gene-trap mutagenesis identifies mammalian genes contributing to intoxication by *Clostridium perfringens* ε-toxin. PLoS ONE.

[84] Rumah K.R., Ma Y., Linden J.R., Oo M.L., Anrather J., Schaeren-Wiemers N., Alonso M.A., Fischetti V.A., McClain M.S., Vartanian T. (2015). The myelin and lymphocyte protein MAL is required for binding and activity of *Clostridium perfringens* ε-toxin. PLoS Pathog..

[85] Knapp O., Maier E., Benz R., Geny B., Popoff M.R. (2009). Identification of the channel-forming domain of *Clostridium perfringens* Epsilon-toxin (ETX). Biochim. Biophys. Acta.

[86] Nestorovich E.M., Karginov V.A., Bezrukov S.A. (2010). Polymer partitioning and ion selectivity suggest asymmetrical shape for the membrane pore formed by epsilon toxin. Biophys. J..

[87] Robertson S.L., Li J., Uzal F.A., McClane B.A. (2011). Evidence for a prepore stage in the action of *Clostridium perfringens* epsilon toxin. PLoS ONE.

[88] Soler-Jover A., Dorca J., Popoff M.R., Gibert M., Saura J., Tusell J.M., Serratosa J., Blasi J., Martín-Satué M. (2007). Distribution of *Clostridium perfringens* epsilon toxin in the brains of acutely intoxicated mice and its effect upon glial cells. Toxicon.

[89] Gil C., Dorca-arévalo J., Blasi J. (2015). *Clostridium perfringens* epsilon toxin binds to membrane lipids and its cytotoxic action depends on sulfatide. PLoS ONE.

[90] Hunter S.E., Clarke I.N., Kelly D.C., Titball R.W. (1992). Cloning and nucleotide sequencing of the *Clostridium perfringens* epsilon-toxin gene and its expression in *Escherichia coli*. Infect. Immun..

[91] Dorca-Arévalo J., Soler-Jover A., Gibert M., Popoff M.R., Martín-Satué M., Blasi J. (2008). Binding of ε-toxin from *Clostridium perfringens* in the nervous system. Vet. Microbiol..

[92] Dorca-Arévalo J., Pauillac S., Díaz-Hidalgo L., Martín-Satué M., Popoff M.R., Blasi J. (2014). Correlation between in vitro cytotoxicity and in vivo lethal activity in mice of epsilon toxin mutants from *Clostridium perfringens*. PLoS ONE.

[93] Dorca-Arévalo J., Martín-Satué M., Blasi J. (2012). Characterization of the high affinity binding of epsilon toxin from *Clostridium perfringens* to the renal system. Vet. Microbiol..

[94] Chandran D., Naidu S.S., Sugumar P., Rani G.S., Vijayan S.P., Mathur D., Garg L.C., Srinivasan VA. (2010). Development of a recombinant epsilon toxoid vaccine against enterotoxemia and its use as a combination vaccine with live attenuated sheep pox virus against enterotoxemia and sheep pox. Clin. Vaccine Immunol..

[95] Li Q., Xin W., Gao S., Kang L., Wang J. (2013). A low-toxic site-directed mutant of *Clostridium perfringens* ε-toxin as a potential candidate vaccine against enterotoxemia. Hum. Vaccine Immunother..

[96] Yao W., Kang J., Kang L., Gao S., Yang H., Ji B., Li P., Liu J., Xin W., Wang J. (2016). Immunization with a novel *Clostridium perfringens* epsilon toxin mutant rETX^Y196E^-C confers strong protection in mice. Sci. Rep..

[97] Alimolaei M., Golchin M., Daneshvar H. (2016). Oral immunization of mice against *Clostridium perfringens* epsilon toxin with a *Lactobacillus casei* vector vaccine expressing epsilon toxoid. Infect. Genet. Evol..

[98] Souza A.M., Reis J.K.P., Assis R.A., Horta C.C., Siqueira F.F., Facchin S., Alvarenga E.R., Castro C.S., Salvarani F.M., Silva R.O.S. (2010). Molecular cloning and expression of epsilon toxin from *Clostridium perfringens* type D and tests of animal immunization. Genet. Mol. Res..

[99] Cole A.R., Gibert M., Popoff M.R., Moss D.S., Titball R.W., Basak A.K. (2004). *Clostridium perfringens* ε-toxin shows structural similarity to the pore-forming toxin aerolysin. Nat. Struct. Mol. Biol..

[100] Pelish T.M., McClain M.S. (2009). Dominant-negative inhibitors of the *Clostridium perfringens* ε-toxin. J. Biol. Chem..

[101] Maassen C.B.M., Laman J.D., Bak-glashouwer M.J.H., Tielen F.J., Holten-Neelen J.C.P.A., Hoogteijing L., Antonissen C., Leer R.J., Pouwels P.H., Boersma W.J.A. (1999). Instruments for oral disease-intervention strategies: Recombinant *Lactobacillus casei* expressing tetanus toxin fragment C for vaccination or myelin proteins for oral tolerance induction in multiple sclerosis. Vaccine.

[102] Goswami P.P., Rupa P., Prihar N.S., Garg L.C. (1996). Molecular cloning of *Clostridium perfringens* epsilon-toxin gene and its high level expression in *E. coli*. Biochem. Biophys. Res. Commun..

[103] Miyata S., Minami J., Tamai E., Matsushita O., Shimamoto S., Okabe A. (2002). *Clostridium perfringens* ε-toxin forms a heptameric pore within the detergent-insoluble microdomains of Madin-Darby canine kidney cells and rat synaptosomes. J. Biol. Chem..

[104] Zekarias B., Mo H., Curtiss R. (2008). Recombinant attenuated *Salmonella enterica* serovar typhimurium expressing the carboxy-terminal domain of alpha toxin from *Clostridium perfringens* induces protective responses against necrotic enteritis in chickens. Clin. Vaccine Immunol..

[105] Jiang Z., De Y., Chang J., Wang F., Yu L. (2014). Induction of potential protective immunity against enterotoxemia in calves by single or multiple recombinant *Clostridium perfringens* toxoids. Microbiol. Immunol..

[106] Gil L.A.F., Cunha C.E.P., Moreira G.M.S.G., Salvarani F.M., Assis R.A., Lobato F.C.F., Mendonça M., Dellagostin O.A., Conceição F.R. (2013). Production and evaluation of a recombinant chimeric vaccine against *Clostridium botulinum* neurotoxin types C and D. PLoS ONE.

[107] Conceição F.R., Moreira A.N., Dellagostin O.A. (2006). A recombinant chimera composed of R1 repeat region of Mycoplasma hyopneumoniae P97 adhesin with *Escherichia coli* heat-labile enterotoxin B subunit elicits immune response in mice. Vaccine.

[108] Marchioro S.B., Fisch A., Gomes C.K., Galli V., Haesebrouck F., Maes D., Dellagostin O., Conceição F.R. (2014). Local and systemic immune responses induced by a recombinant chimeric protein containing *Mycoplasma hyopneumoniae* antigens fused to the B subunit of *Escherichia coli* heat-labile enterotoxin LTB. Vet. Microbiol..

[109] Terpe K. (2006). Overview of bacterial expression systems for heterologous protein production: From molecular and biochemical fundamentals to commercial systems. Appl. Microbiol. Biotechnol..

[110] Khorasani A., Madadgar O., Soleimanjahi H., Keyvanfar H., Mahravani H. (2016). Evaluation of the efficacy of a new oil-based adjuvant ISA 61 VG FMD vaccine as a potential vaccine for cattle. Iran. J. Vet. Res. Shiraz Univ..

[111] Aucouturier J., Dupuis L., Ganne V. (2001). Adjuvants designed for veterinary and human vaccines. Vaccine.

[112] Cloete M., Dungu B., van Staden L., Ismail-Cassim N., Vosloo W. (2008). Evaluation of different adjuvants for foot-and-mouth disease vaccine containing all the SAT serotypes. Onderstepoort J. Vet. Res..

[113] Sumithra T.G., Chaturvedi V.K., Siju S.J., Susan C., Rawat M., Rai A.K., Sunita S.C. (2013). Enterotoxaemia in goats—A review of current knowledge. Small Rumin. Res..

[114] Bernáth S., Fábián K., Kádár I., Szita G., Barna T. (2004). Optimum time interval between the first vaccination and the booster of sheep for *Clostridium perfringens* type D different authors suggest very different time intervals for the revaccination of sheep, even when similarly adjuvanted vaccines are used again. Acta Vet. BRNO.

